# An Analysis on Sensor Locations of the Human Body for Wearable Fall Detection Devices: Principles and Practice

**DOI:** 10.3390/s16081161

**Published:** 2016-07-25

**Authors:** Ahmet Turan Özdemir

**Affiliations:** Department of Electrical and Electronics Engineering, Erciyes University, Kayseri 38039, Turkey; aturan@erciyes.edu.tr; Tel.: +90-352-207-6666 (ext. 32233)

**Keywords:** fall detection, wearable motion sensors, sensor placement, elderly people, machine learning techniques, classification, feature extraction and reduction

## Abstract

Wearable devices for fall detection have received attention in academia and industry, because falls are very dangerous, especially for elderly people, and if immediate aid is not provided, it may result in death. However, some predictive devices are not easily worn by elderly people. In this work, a huge dataset, including 2520 tests, is employed to determine the best sensor placement location on the body and to reduce the number of sensor nodes for device ergonomics. During the tests, the volunteer’s movements are recorded with six groups of sensors each with a triaxial (accelerometer, gyroscope and magnetometer) sensor, which is placed tightly on different parts of the body with special straps: head, chest, waist, right-wrist, right-thigh and right-ankle. The accuracy of individual sensor groups with their location is investigated with six machine learning techniques, namely the *k*-nearest neighbor (*k*-NN) classifier, Bayesian decision making (BDM), support vector machines (SVM), least squares method (LSM), dynamic time warping (DTW) and artificial neural networks (ANNs). Each technique is applied to single, double, triple, quadruple, quintuple and sextuple sensor configurations. These configurations create 63 different combinations, and for six machine learning techniques, a total of 63 × 6 = 378 combinations is investigated. As a result, the waist region is found to be the most suitable location for sensor placement on the body with 99.96% fall detection sensitivity by using the *k*-NN classifier, whereas the best sensitivity achieved by the wrist sensor is 97.37%, despite this location being highly preferred for today’s wearable applications.

## 1. Introduction

Wearable device applications have gained incredible popularity in many areas of daily life, such as health [1], entertainment [2], communication [3], rehabilitation [4] and education [5]. On the other hand, thanks to the reduced service cost of communication networks, users can easily access the Internet at very reasonable prices. These advances in wearable devices and Internet technologies have resulted in today’s wearable and Internet of Things (IoT) patent wars. Wearable fall detection devices are one of the most popular fields in both academia and industry. This is because falls are a serious and common cause of morbidity and mortality among elderly people [6]. More than one third of elderly people, aged 65 or older, experienced at least one fall each year [7]. Fall detection devices can create fall alarms immediately and alert relevant persons for assistance. Immediate aid after a fall reduces the costs of treatment and the hospital length of stay. If a falling person stays unattended for a long time, physical and psychological complications can be observed. Physical complications depend on the severity of the injury and cover a reasonably broad spectrum from simple scratches and contusions to mortal (fatal) brain damage and hip fractions [8,9]. Psychologically, falls induce fear of falls and other physical activities; this feeling makes people more likely to fall again. Staying time on the floor after a fall has great importance in physiological complications; increasing time has deeper effects on subjects, like social isolation [7]. 

There are many techniques that are used to detect falls, such as camera systems and smart grounds; however, wearable systems are known as the most preferred solution [1,4,10,11,12]. This is because other systems have privacy problems and/or force people to live in a restricted area. Wearable fall detection systems produce high accuracy and improve mobility. These advantages make wearable device applications more preferable than the others [11,13]. Wearable fall detection devices can be divided into two main groups: user-manipulated and automatic systems [1,4]. User-manipulated systems basically have a panic button, and when the subject experiences a fall, he/she activates the button to create a fall alarm. In this way, relevant persons can be informed about the fall. User-manipulated fall detection systems are easy to use and inexpensive, but these systems are non-functional during loss of consciousness when first aid is needed the most by the user. Therefore, a wearable fall detection system should be automatic.

Different wearable sensor-device applications for fall detection exist in the literature and on the market. However, it is still impossible to compare the accuracies of these approaches and devices. There are many reasons for this; the first is that researchers do not use common activity and fall sets for evaluating system performance. Generally, different studies use different sets of activities and falls that are performed by different subjects to create their own dataset [14,15]. Thus, experimentation and evaluation standards are needed for better comparison. Another problem is the different types of sensors used during the evaluation, such as a combination of accelerometer, gyroscope, magnetometer, barometer or microphone [13,16,17,18,19,20,21,22]. Usage of different decision algorithms to detect falls is also an issue for the comparison of the accuracies of different devices [16]. Depending on the device ergonomics, different body locations are chosen for sensor placement for wearable fall detection devices [14]. There are some works for standardizing activity and fall movement sets [23] and comparing sensor achievements [17]; however, the literature lacks a study that specially focuses on determining the best sensor placement part of the body for single sensor-based solutions. If the most suitable body location for sensor placement is known, fall detection devices and test experiments can be better designed to achieve better accuracy by taking this location into consideration. 

Falls are associated with major risk factors, like chronic illness, disabilities and aging [24]. People in fall risk groups mostly have motion limitations. These risk factors and motion limitations require wearable fall detection devices that are easy to use and wear. Single sensor placement solutions can make fall detection devices easy to wear and use. In the literature, different single sensor positions, such as waist, chest, head, wrist, leg, ankle and arm, are considered with the claim of the best fall detection accuracy; however, different movement datasets with different classification and decision algorithms are used in these works [10]. Therefore, it is impossible to assess the best body locations for fall detection accuracy on the basis of these results.

Bao et al. used five biaxial accelerometers and asked 20 volunteers, 13 males and 7 females, with ages from 17 to 48, with a mean 21.8, to wear these sensors on different parts of their bodies: hip, wrist, ankle, arm and thigh. They distinguished 20 different everyday activities using a decision table, instance-based learning (nearest neighbor), naive Bayes and the decision tree classification algorithm. The best accuracy is achieved with the decision tree algorithm at 84% overall success. They reported that the classification performance is reduced only 3.27% when two biaxial accelerometers are attached to the thigh and wrist [21]. This work proves that reducing the number of sensor nodes from five to two slightly decreases the accuracy. 

Kangas et al. defined nine sets of fall actions performed by three volunteers (one 38 year-old female and two males that were 42 and 48 years-old, respectively). Activities of daily living (ADLs) are performed by the female and the 42 year-old male. During the simulated falls and ADLs, acceleration of the body is measured simultaneously from the waist, head and wrist with a triaxial accelerometer. Waist sensor sensitivity varies from 76% to 97%; head sensor sensitivity varies from 47% to 98%; and lastly, wrist sensor sensitivity varies from 37% to 71%. In this study, the head was found to be the best sensor location for detecting falls [18]. This work gives some ideas about three different body locations, but the diversity of activities and the number of volunteers are not sufficient to give a final decision about defining the best sensor placement location. 

Li et al. aimed to distinguish falls from ADL activities and used a two accelerometer and a gyroscope combination instead of just an accelerometer. They created a dataset using 18 different trials, 5 falls and 13 ADLs, including fall-like activities, with three men in their 20s. Using two different sensor nodes each consisting of a triaxial accelerometer and a triaxial gyroscope on the chest and upper limb, falls from ADLs were recognized with 91% sensitivity [19]. However, this approach uses both chest and limb sensors, and individual sensor performances were not calculated. Therefore, this work does not exactly determine the best sensor placement part of the body. Volunteers and the diversity of trials are also not sufficient.

Atallah et al. discussed the literature about accelerometer-based wearable activity recognition and fall detection systems in terms of sensor placement. They argue that increasing the number of sensor units and the types, such as a combination of accelerometer, gyroscopes, magnetometers, microphone and pressure sensors, increase classification accuracy. Eleven subjects (nine males and two females) wore six triaxial accelerometers on different parts of their bodies (chest, arm, wrist, waist, knee and ankle) and one ear-worn sensor, then performed 15 sets of ADLs. The movement sets used in this work do not contain fall actions. Activities were recorded with battery-powered light-weight boards, and 13 features are extracted from these records. They found that features are highly affected by changes in orientation. *k*-NN and Bayesian classifier algorithms are used to classify activities; however, the optimal sensor location was not determined [20]. 

Shi et al. reported that there is a lack of determining the optimal sensor position for fall detection systems in the literature. They used 17 sensor nodes each consisting of a triaxial (accelerometer, gyroscope and magnetometer), as well as a contact pressure sensor. Thirteen young volunteers (12 males and one female, height in the range 160 cm to 185 cm, weight in the range 55 kg to 85 kg) performed 12 sets of ADLs and 13 sets of falls. Volunteers performed each fall pattern 10 times and each ADL pattern 20 times, and they created a dataset consisting of 3232 records. During the tests, they recorded motions wirelessly with these sensors attached to the thighs, shanks, feet, upper arms, forearms, hands, waist, neck, head and back. They found that the use of sensors on the waist and feet can detect falls with 98.9% sensitivity. The maximum single sensor sensitivity achieved in this study was 95.5% in the waist and upper waist separately using the decision tree classification algorithm [22]. The ratio of female participants is only 1/13; this situation makes the dataset unbalanced and dominated by men’s data. Anatomic differences between genders are very important in terms of sensor orientations and the execution style of individual movements. Another issue about this work is the number of repetitions for each test, because 10 or 20 repetitions are extremely exhausting for a volunteer. These high repetition rates disrupt the naturalness of the activities.

In this work, an activity and fall dataset [13] that covers 2520 records of seven males and seven females was used. These 14 volunteers performed 36 sets of movements, including ADLs (16 sets) and falls (20 sets) with five repetitions. During the tests, the volunteer’s movements were recorded with six sensor units, each with a triaxial sensor (accelerometer, gyroscope and magnetometer), placed on different parts of the body. These locations are head, chest, waist, right wrist, right thigh and right ankle. The main motivation of this work is to determine the best sensor positioning for wearable fall detection devices out of these six locations on the human body. The classification performances of these sensors are investigated with six machine learning techniques, namely the *k*-nearest neighbor (*k*-NN) classifier, Bayesian decision making (BDM), support vector machines (SVM), least squares method (LSM), dynamic time warping (DTW) and artificial neural networks (ANNs). Each technique is applied to single, double, triple, quadruple, quintuple and sextuple sensor configurations. These configurations create 63 different combinations, and for six machine learning techniques, a total of 63 × 6 = 378 combinations is investigated. Although the classification results of these machine learning algorithms are very satisfactory and stable, this work does not mainly aim to present a robust fall detection device or system, but to propose the best sensor location on the human body for wearable fall detection devices. This work is especially focused on sensor positioning effects over fall detection performance of the algorithms and the most comprehensive investigation in the literature in terms of the number of analyzed sensor combinations, employed machine learning algorithms and rates of accuracy and sensitivity for single sensor options.

The rest of this work is organized as follows. In Section 2, the System Design section, information about the dataset and volunteers, sensor specifications, experimental setup, data formation and feature extraction and reduction topics are discussed. In Section 3, the Materials and Methods section, sensor placement combinations on the body, performance metrics and six machine learning algorithms used in this work are described briefly. Section 4, the Results and Discussion section, is the section where the results are presented and the performances of the machine learning algorithms with a total of 378 different sensor configurations are discussed. In the last section, conclusions section, the paper is finalized with the description of the possible future works. 

## 2. System Design 

**Figure 1:**
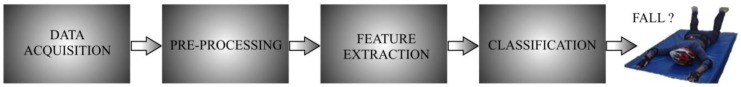
Fall detection system design block diagram.

There are four main tasks that exist in this work, as shown in Figure 1. The first one is the data acquisition task, and in this step, a huge dataset has been created with 14 volunteers including 2520 records. The name of the second task is preprocessing, and in this step, meaningful data have been formed from the huge dataset, which was already created previously. The feature extraction task is the third step, and in this step, the raw dataset created in the preprocessing step has been reduced in size by using the feature extraction and dimension reduction techniques. The last step of the fall detection system is the classification process. The classification task uses dimensionally-reduced features and gives a binary decision about fall events using machine learning techniques. 

### 2.1. Dataset, Volunteers and Tests

The first step in the design of a robust fall detection system is to use a comprehensive movement dataset, including ADLs that are mostly confused with falls, such as lying in bed, jogging, stumbling, etc. In general, researchers create their own datasets. Because of this, fall detection performance of different platforms cannot be compared. In this work, ADL and fall movements are adopted from [25] as a trial, and the trial protocol was approved by the Erciyes University Ethics Committee (Approval Number 2011/319). Volunteers received written informed consent and oral information about the tests. The seven healthy men aged 24 ± 3 years old, weighing 67.5 ± 13.5 kg and with a height of 172 ± 12 cm are named 101, 102, 103, 104, 106, 107 and 108. The seven healthy women aged 21.5 ± 2.5 years old, weighing 58.5 ± 11.5 kg and with a height 169.5 ± 12.5 cm are named 203, 204, 205, 206, 207, 208 and 209 (see Table 1). 

The trial used in this work consists of 20 falls and 16 ADLs. These 36 movements are performed by 14 young subjects with five repetitions each. The resulting dataset covers 2520 records (36 tests × 14 subjects × 5 repeats = 2520 records) each 15 s long on average. Table 2 summarizes movements in terms of falls and ADLs. Each movement is labeled with a number; 8XX defines falls, and 9XX defines ADLs. A record contains a volunteer definition, a movement definition and repetition time. 102_903_3 is an example of a record label and it refers to the male volunteer-102 performing the sit bed activity (903) of the third (3) round, as defined in Table 2.

The movement database consists of 2520 uniquely-named records of complex inertial, magnetic, atmospheric pressure sensor and microphone data. Fall movements in the table are commonly observed in real life, and ADLs contain movements that can easily be confused with falls. Using this standard trial set allows comparability and robustness for a classification system. This movement set helps in designing robust classifiers, because falls and activity types are sufficient to simulate real-life conditions. It also helps in comparing the results with other works, because the movements are standard.

### 2.2. Sensor Specifications

**Figure 2:**
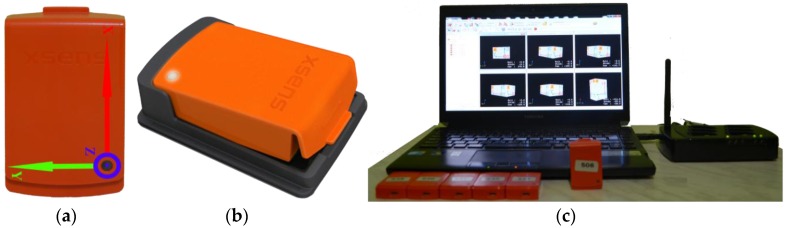
(**a**) MTw sensor unit; (**b**) Sensor unit with housing [26]; (**c**) Wireless data acquisition system.

In this work, six three-DOF orientation tracker MTw units (see Figure 2), produced by Xsens Technologies, are used [26]. Each unit has a triaxial gyroscope that can sense in the range of ±1200°/s angular velocities, a triaxial accelerometer that can sense in the range of ±160 m/s^2^ acceleration, a triaxial magnetometer that can sense earth magnetic fields in the range of ±1.5 Gauss and a pressure sensor that can sense atmospheric pressure in the range of 300 to 1100 mBar. Inertial and magnetic data in three axis (*x*, *y*, *z*) were acquired at a 25-Hz sampling rate, each test lasting about 15 s on average.

Although inertial and magnetic sensor units are designed for ambulatory and motion tracking purposes, generally in restricted laboratory environments, they have successfully been used for indoor, as well as outdoor movement analysis and started to gain increased popularity in the study of human motion analysis [27,28]. 

Atmospheric pressure and microphone data were not used in the feature extraction and classification processes because changes on the pressure sensor’s output during movements did not provide meaningful information. In addition, there was too much noise around, and the microphone recorded unwanted sounds with the voices and movements during the tests. Preventing noise in the restricted test area is not a solution because noise will be present in real life, as well. Therefore, in order to create a robust classifier, acquired data must reflect a real environment. Works in the literature mostly suffer from laboratory-based setups [11,13]. This is the answer to the question of why there are many works in the literature that have very good fall detection sensitivities, but there is no off-the-shelf product on the market. In this work, test scenarios need to be realistic and compatible with real-life conditions. 

Inertial and magnetic sensors that are used in smartphones and other wearable devices may not be as sensitive and give as broad a range of motion data as MTw units do. However, the performances of different sensor units placed at the same location on the human body as proposed in this work should be similar to the sensors employed in this study. This works aims to determine the optimum body locations for general wearable fall detection devices. Therefore, it is believed that the results achieved in this work can be observed with different sensors with the same setup. 

### 2.3. Experimental Setup

**Figure 3:**
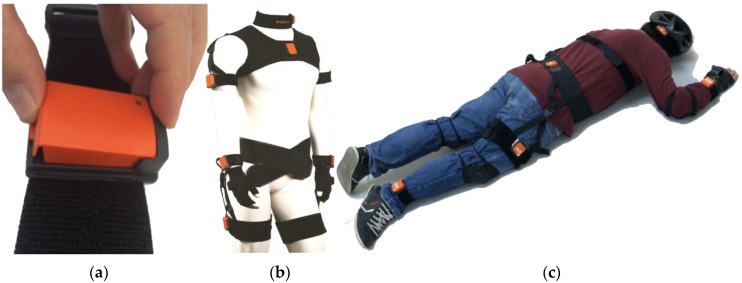
(**a**) MTw unit housing on a strap; (**b**) Strap set on mannequin [26]; (**c**) Sensors placement on the subject’s body.

Six MTw sensor units are placed on different parts of the subject’s body with a special strap set. Each strap has sensor housing; thus, the MTw sensor units are easily attached to and detached from these straps. A special strap with sensor housing for the wrist is shown in Figure 3a. Extra foam covers are wrapped around all sensor units in order to protect them from direct impact on the ground. Because the sensor’s case is made of very tough plastic material, this hard surface increases the impact of shock in the case of a direct hit. The human body is relatively soft, and such an amount of exaggerated acceleration data cannot be observed on it. 

Each strap is specially designed for individual body parts (see Figure 3b). In this work, the head, chest, waist, right wrist, right thigh and right ankle were chosen as specific sensor locations (see Figure 3c). Each sensor unit is marked with a letter as follows: A-head, B-chest, C-waist, D-right wrist, E-right thigh and F-right ankle. It is very important to attach sensors to the subject’s body tightly in order to collect body movements correctly. MTw sensor units connect to a computer over ZigBee. Wireless data acquisition systems let the user perform movements more naturally than cabled systems. Elaborate precautions were taken to prevent volunteers from injuries as part of the ethics committee approval. First of all, volunteers were asked to wear a helmet, elbow pads, knee and wrist guards. These protective clothes guard the body parts from dangerous shock. Lastly, fall movements are performed on a soft crash mat to reduce the shock effect on the body.

Current microelectromechanical systems (MEMS)-based inertial and magnetic sensors are typically light and thin; thus, they are preferred to be used as accessories or clothes. This work chose sensor locations to be the same places where accessories or daily wearables can be worn, such as the head (A) with hat, glasses and earrings, chest (B) with underwear, necklace and brooch, waist (C) with belt and waistband, wrist (D) with watch, gloves and armband, thigh (E) with pocket and ankle (F) with socks, shoes and boots [29]. Strap sets used in this work are suitable for these locations, and they perfectly attached sensors to the body, as shown in Figure 3b. Furthermore, thigh, waist, chest, wrist and head are important locations for collecting vital biomedical signals, such as EMG, EKG and EEG or heat and perspiration. Vital body parameters can be gathered with custom-designed fall detection devices and sent to remote monitoring services together with motion data. 

The movements on one side of the body have opposite effects of the movements of similar pattern on the other side of the body [30]. Because of this biomechanical symmetry property, the sensors were attached to the ankle, thigh and waist only on the right side of the body. Moreover, decreasing the number of sensors used on outer limbs also reduces the computational complexity of classification algorithms by using one side of the body. 

### 2.4. Data Formation

**Figure 4:**
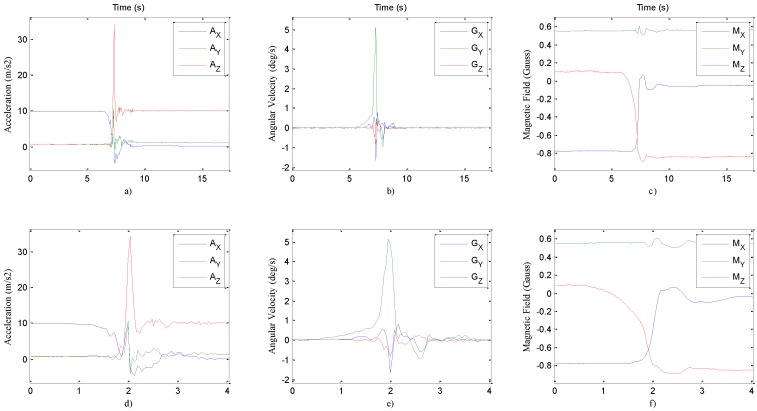
These six graphics belong to the waist sensor and show the first repetition of five 901-Front Lying fall actions performed by Volunteer 203 (203_901_1). The top three graphic ((**a**) to (**c**)) are saved with 430 samples (more than 17 s-long raw data record, sampled at 25 Hz), and the bottom three graphics ((**d**) to (**f**)) are reduced to a 101 sample (nearly 4 s-long shortened data) record.

Xsens’ Awinda Station (Figure 2c) reads six MTw units with RF connection and transfers the data to a PC with a USB interface. This unit is controlled by Xsens’ MT Manager Software, and this software creates six comma-separated-values files simultaneously in a single test. Each data file comprises 10 columns and 25 lines per second; average line counts are 15 s × 25 Hz = 375 lines. Columns are Counter, $A_{x},\;A_{y},\;A_{z},\;G_{x},\;G_{y},\;G_{z},\;M_{x},\;M_{y},\;\;\text{and}\;M_{z}$. Counter is a kind of time stamp and counts up with each sample; counter data are used to check synchronization and missed data. *A*, *G*, and *M* are abbreviations of acceleration, gyroscope, and magnetometer sensors’ data respectively. x, y, and z represent perpendicular axes please see Figure 4. At the end of the complete trial, 15,120 files, consisting of 36 movements (Table 2), were created (14 volunteers × 36 tests × 5 repeats × 6 files = 15,120). In order to reduce the dimension of this huge dataset, 15 s-long frames are cut into 4 s-long frames. A 4-s frame consists of two 2-s frames that are around the maximum total acceleration (TA) of the waist sensor. TA is given in Equation (1):

(1) $$TA=\sqrt{{A_{x}}^{2}+{A_{y}}^{2}+{A_{z}}^{2}}$$

TA is a vector consisting of *m* values of average acceleration along the *x*, *y* and *z* axes, which are, respectively, $A_{x},\;A_{y},\;A_{z}$. A test lasts around 15 s; therefore the *m* value is approximately 375 (15 s × 25 Hz). The first 50 (2 s × 25 Hz) and the last 50 (2 s × 25 Hz) elements of a test record are not considered; outliers are eliminated, because these sections cover preparation and completion of an activity; therefore, high acceleration values in these phases are not meaningful. The maximum acceleration value in the TA vector was searched in the remaining 275 values, and a 4 s-long vector is constructed using two 2 s-long samples around the maximum value of TA vector. A 2 s sample consists of 50 elements (2 s × 25 Hz); therefore, two 2 s frames around the maximum TA value contain 101 elements (50 samples + max. TA value + 50 samples) (Figure 4). The same data reduction procedure is applied to the remaining five sensor units using the exact time index stamped with the Counter column in a record. A record contains acceleration, rate of turn and Earth magnetic field values in three axes.

In this manner, six 101 × 9 (101 lines × 9 columns) arrays of data are created from a single repetition of a test with a sensor unit, given in Table 2. Each column of data defines acceleration, angular velocity or Earth magnetic field in the *x*, *y* or *z* axis. A column is represented by an *N* × 1 vector $d=\;{\left[d_{1},\;d_{2},\;\ldots ,d_{N}\;\right]}^{T}$, where *N* = 101. The first three features extracted from the vector *d* are the minimum, maximum and mean values. The second three features are variance, skewness and kurtosis. The other eleven features are the autocorrelation sequence consisting of the first 11 values. The last ten features are the first five peaks of the discrete Fourier transform (DFT) of the column with the corresponding frequencies. In this work, six types of features were utilized, and five of them are statistical features (mean, variance, skewness, kurtosis and autocorrelation). Four second-long signal segments were assumed to be the realization of an ergodic process. Therefore, time averages were used instead of ensemble averages. However, in order to prevent features from being spatial domain dependent, DFT features were used, as well. In this work, discrete cosine transform and the total energy of the signal were also considered. However, since extra coefficients and features extend the size of the feature vector, extra features, such as the total energy, cosine transform or wavelet transform coefficients, were not used. If the classification results of this work were not satisfactory, extra features would be used to improve the accuracies of machine learning algorithms for fall detection.

(2) $$mean(d):\mu =\frac{1}{N}\sum_{i=1}^{N}d_{i}$$

(3) $$\mathrm{var}iance(d):\sigma^{2}=\frac{1}{N}\sum_{i=1}^{N}(d_{i}-\mu {)}^{2}$$

(4) $$skewness(d):\frac{1}{N\sigma^{3}}\sum_{i=1}^{N}(d_{i}-\mu {)}^{3}$$

(5) $$kurtosis(d):\frac{1}{N\sigma^{4}}\sum_{i=1}^{N}(d_{i}-\mu {)}^{4}$$

(6) $$autocorrelation(d):R_{ss}(\Delta )=\frac{1}{N-\Delta }\sum_{i=0}^{N-\Delta -1}(d_{i}-\mu )(d_{i-\Delta }-\mu ),\Delta =0,1,...,N-1$$

(7) $$DFT(k)=\sum_{i=0}^{N-1}d_{i}e^{-j2\pi ki/N},k=0,1,...,N-1$$

**Figure 5:**
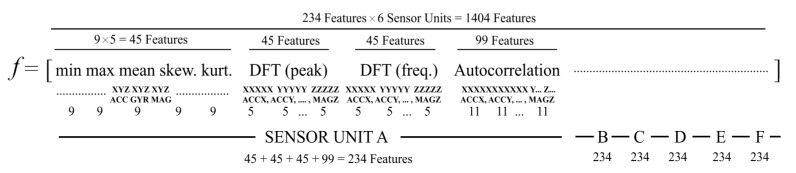
Feature vector formation.

Here, $d_{i}$ represents the *i*-th element of discrete *d* vector. *µ* is the mean, and *σ* is the standard deviation of *d*. $R_{ss}\left(\Delta \right)$ is the unbiased autocorrelation discrete-time sequence of *d*, and *DFT(k)* is the *k*-th element of *N* point DFT. Here, the maximum, the minimum, mean, skewness and kurtosis features are all scalar values. The autocorrelation feature has eleven values, and the DFT feature has ten values. Each sensor unit (MTw) is triaxial, $\left(A_{x},\;A_{y},\;A_{z},\;G_{x},\;G_{y},\;G_{z},\;M_{x},\;M_{y},\;M_{z}\right)$; therefore, in total, nine signals are recorded from a unit. Since there are six sensor units, 54 signals (9 signals × 6 sensors) exist, and a total of 270 features (54 signal × 5 features) are created for the first five scalar features. Similarly, 270 features are created for the first five DFT peak values, and the other 270 features are created for the five respective frequencies. Lastly, 594 features (11 × 9 × 6) are created for 11 autocorrelation sequences. The feature formation process resulted in a feature vector of dimension 1404 × 1 (270 + 270 + 270 + 594) for each test of the 4-s signal segments. Feature vector formation is shown in Figure 5. This feature vector was created for a single test. However, in this work, there are 2520 movements, including 1400 falls (14 volunteers × 20 falls × 5 repetitions) and 1120 ADLs (14 volunteers × 16 ADLs × 5 repetitions). Therefore, a feature set dimension of a 1404 × 2520 vector array is generated from 2520 features extracted from the movements.

### 2.5. Feature Extraction and Dimensional Reduction

The resulting feature vector is quite big in size (1404 × 1); this situation increases the computational complexity of the classifiers, both in the training and testing phases. Therefore, the size of the feature vector (see Figure 5) needs to be reduced in order to simplify the computational process. Because each element of a feature vector does not have an equal contribution in defining individual movement, each movement has a different signal pattern. Therefore, the principal component analysis (PCA) technique is used as the dimension reduction method. PCA is the most traditional and efficient dimension reduction technique and transforms the original variables f(1),f(2),…,f(m) into a smaller group of different variables x(1),x(2),…,x(p), where p≤m [31] and *m* is 1404. A single feature vector is given as below:

(8) $$f_{k}={\left[f{\left(1\right)}_{k}\;f{\left(2\right)}_{k}\ldots \;f{\left(1404\right)}_{k}\right]}^{T}$$

Here, *k* = 2520, and the raw feature set *F* is defined by a matrix of 2520 × 1404 in size.

(9) $$F=\;\left[\;f_{1}\;f_{2}\ldots \;f_{2520}\right]$$

$\bar{f}$ defines the mean of an individual feature vector, and it is calculated for each feature vector as below. Here, *n* equals the number of elements in a feature vector, which is 1404 elements.

(10) $$\bar{f}=\frac{1}{1404}\sum_{n=1}^{1404}f(n)$$

$P_{1},P_{2},\ldots ,P_{i}$ are principal components (PCs) and calculated by the following formula. Here, *C_f_* is the covariance matrix of a feature vector, and the first 30 leading eigenvectors of the covariance matrix give principal components. The first principal component has the largest possible variance and each succeeding principal in turn.

(11) $$C_{f}=\frac{1}{1403}\sum_{n=1}^{1404}\{\left(f(n)-\bar{f}\right){\left(f(n)-\bar{f}\right)}^{T}\}$$

An orthogonal basis set is produced by using symmetric *P_i_* eigenvectors and λ*_i_* eigenvalues from the *C_f_* covariance matrix. Therefore, PCs are orthogonal, as well.

(12) $$C_{f}P_{i}=\lambda_{i}P_{i}\;,i=1,2,...,30$$

However, λ*_i_* eigenvalues need to be calculated first in order to find *P_i_* eigenvectors. Here, *I* is the identity matrix.

(13) $$\det\left(C_{f}-\lambda I\right)=0$$

This process shows how principal components (PCs) are calculated for a single raw feature vector. The last reduced size of the feature set is calculated by repeating this process 2520 times. The initial raw feature data vector was quite big (1404 × 1), and this situation resulted in a computationally complex process for distinguishing of falls from ADLs. Therefore, the raw feature vector, normalized first between zero and one, was reduced from 1404 to 30 elements using PCA. In this work, 30 PCs are used because 30 PCs constitute 72.38% of the total variance of the original data (1404-long feature vector), and this ratio represents much of the variability of the raw feature vector.

## 3. Materials and Methods

Six inertial and magnetic sensors create 2^6^ (= 64) different sensor placement combinations on the body, as given in Table 3. However, one of these combinations is not applicable, as shown in the first line of the table, since it refers to the sensorless configuration. The rest of the 63 possible configurations are evaluated with six different machine learning techniques, and the fall detection accuracies of these configurations are specifically calculated for each. Sixty three different sensor placement combinations with six machine learning techniques create a total of 63 × 6 = 378 combinations. Therefore, each of these combinations is scored with its individual accuracy in the MATLAB environment. In this way, one can decide which body part is the most convenient for sensor placement in fall detection applications. Machine learning techniques used in this work are discussed briefly in this section.

Since wearable devices are mobile and battery powered, the power consumption of the system must be considered [32]. Another important issue about wearable fall detection systems that needs to be consider is adoption by the elderly population [33]. Elderly people are in the main fall risk group; most of them have motion limitation and are not familiar with current technology. Therefore, technologically, a fall detection system is required to be simple to use. These requirements force the designer to find the optimum solution. In this work, the main motivation is to reduce the number of sensor nodes attached to the user body. When the number of sensor nodes is reduced, an elderly person uses the system more easily, and the power consumption of the system is reduced. The required computational power in both feature extraction and decision steps also decreases with the reduction of sensor units. For example, in the feature extraction step, the feature vector size is 1404 × 1 with 6 sensors, but this feature vector size with a single sensor is only 234 × 1. This reduction in the size of the feature vector also reduces the computational complexity in the feature extraction step. Another important advantage is about PCs, since classification algorithms use the first 30 PCs of the raw feature vector, and these 30 PCs are much more descriptive of the new raw data. For example, while the largest 30 eigenvalues, in other words 30 PCs, constitute 72.38% of the total variance of PCs of a 1404 × 1 sized feature vector, the same amount of PCs constitutes 96.63% of the total variance of the principal components of a 234 × 1 sized feature vector and account for almost all of the variability of the data. This improves classification performances, because the reduced size of the feature vector with the PCA method can make the raw data much more descriptive.

The designed fall detection system in this work produces a binary decision about the fall event, whether it occurred or not. The performances of machine learning techniques, which are employed in this work, are compared in terms of accuracy, sensitivity and specificity. In order to determine these performance parameters, four possibilities that a binary classifier can encounter need to be considered. The first possibility is called true positive (*TP*), and in this case, a fall occurs and the algorithm detects it. In the second case, a fall does not occur, and the algorithm does not produce a fall alert. This possibility is called a true negative (*TN*). *TN* and *TP* cases are truly given decisions by the algorithm. Wrong decisions given by algorithm are annotated with false labels. The third case refers to false positive (*FP*), and in this case, a fall does not occur, but the algorithm creates a fall alert; this case is also called a false alarm. The most dangerous and unwanted case is called a false negative (*FN*), and in this case, a fall occurs, but the algorithm does not detect it. This case is also called a missed fall.

The binary classifier’s parameters can be formulated using the definitions given above. The rate of truly classified falls to all falls is called the sensitivity (*Se*). In other words, *Se* is a parameter defining how successfully the algorithm senses falls. *Se* measures the proportion of positives that are correctly identified as falls and indicates how well the algorithm predicts the falls.

(14) $$Se=\;\frac{Truly\;Classified\;Falls}{All\;Falls\;\left(Positives\right)}\times 100\;=\;\frac{TP}{TP+FN}\;\times 100$$

Specificity (*Sp*) measures the proportion of negatives that are correctly identified ADLs. *Sp* refers to the ability of the algorithm to correctly identify ADLs and indicates how well the algorithm predicts the ADLs.

(15) $$Sp=\;\frac{Truly\;Classified\;ADLs}{All\;ADLs\;\left(Negatives\right)}\times 100\;=\;\frac{TN}{TN+FP}\;\times 100$$

Accuracy (*Acc*) is expected to measure how well the algorithm predicts both *Se* and *Sp*. *Acc* is derived from *Se* and *Sp*, as well.

(16) $$Acc=\;\frac{Truly\;Given\;Decisions}{All\;Decisions}\times 100\;=\;\frac{TP+TN}{TP+TN+FP+FN}\;\times 100=\;\frac{Se+Sp}{2}\;$$

Hence, a good binary classifier is expected to have high scores for all three factors, *Se*, *Sp* and *Acc*. However, specifically for the fall detection problem, the success of the algorithm is mostly dependent on the frequency of *FN* decisions. False alarms, *FP*, can be ignored by the user, as this fault is not considered an important problem. However, a missed fall, *FN*, is a serious mistake for the algorithm, and for a reliable binary classifier, *FN* is expected to be 0. For any classifier, there is a tradeoff between *Sp* and *Se*, and this relationship can be formulated as below.

(17) FP ratio=1−Sp

(18) FN ratio=1−Se

In this work, it is aimed to improve the *Acc* parameter with a 100% Se success rate, because the reliability of the algorithm increases with increased sensitivity. In some cases, loss of consciousness and movement can be observed depending on the fall. On the other hand, loss of consciousness and movement can trigger a fall, as well. Hence, in these scenarios, if the algorithm misses the fall action, the person who experienced the fall will be alone without help when he/she needs it the most. Therefore, fall detection systems must have very high *Se* rates, and there is no tolerance for missing falls. However, false alarms, *FPs*, produced by the algorithm will confuse and occupy the system unnecessarily and need to be avoided, as well. 

Recent work by the author [13] reports successfully distinguished fall actions from real-world ADLs; many of them are high impact activities that may easily be confused with falls. In that work, 6 sensor units each have a three-axis accelerometer, gyroscope and magnetometer fitted to the subjects’ body at six different positions, and falls are distinguished falls with 100% *Se* and 99.8% *Sp* [13]. However, this work differs from the previous one in that it is aimed to make the system easier to use by reducing the number of sensor nodes while keeping the *Se* rate at an acceptable level. Either in the training or testing phases, the dataset is randomly split into *p =* 10 equal partitions, and *p*-fold cross validation is employed. Nine of the ten partitions, *p* − 1, were used in training, and the rest of the one subset was used in the testing phases. For the purpose of each record in the dataset getting a chance for validation, the training and testing partitions crossed over in *p* successive rounds. This process is applied not only for each record having a contribution on both the training and validation stages, but also to avoid the approximation errors that may occur due to the unbalanced number of falls or ADLs among rounds.

### 3.1. The k-Nearest Neighbor Classifier 

Basically, the *k*-NN method classifies a test object by finding the closest training object(s) to it [34]. The binary decision is made by using nearest neighbors *k*, where *k >* 0, and it is a user-defined value; majority voting determines the class decision. However, because the *k*-NN algorithm is sensitive to local dataset composition, a proper *k* value should be defined specifically for the individual problem. There is a tradeoff between sensitivity and robustness. Therefore, determining the *k* value is critical. For example, a larger *k* value reduces the sensitivity by increasing the bias, whereas smaller *k* values produce less stable results by increasing the variance. This explains why the correct *k* value depends on the local data. The *k* values between 1 and 15 have been tried, and the best result is obtained with *k* = 7. 

### 3.2. Bayesian Decision Making 

BDM is a robust algorithm and frequently preferred in statistical pattern classification problems. In this work, likelihood in BDM is defined by the normal density discriminant function, and the estimation of the class decision is given by using the maximum likelihood indexes for a given test vector *x*. The function parameters are the mean of the training vectors *µ* and the covariance matrix of the training vectors *C* for each class [34]. Since the mean vector and covariance matrix are calculated using the training records of the two classes, these values are constant for each fold. Maximum likelihood is searched, and the class decision is given for each test vector as follows:

(19) $${ℒ}_{\left(class\;i\right)}=\;-\frac{1}{2}\{{(x-\mu_{i})\;}^{T}C_{i}^{-1}\left(x-\mu_{i}\right)+\text{log}\left[\text{det}\left(C_{i}\right)\right]\}\;i\;=\;1,2$$

### 3.3. Support Vector Machines

SVM is a very promising classification algorithm; however, it does not guarantee the best accuracy for all kinds of problems. The initial set of coefficients and kernel models also affect the classification accuracy. $\left(x_{j},l_{j}\right),\;$*j* = 1,…,*J* is the training data with the length of *J*, where *x_j_* ∈ ℝ^N^. *lj* is the class labels *lj* ∈ {1, −1}; there are two classes that exist: falls and ADLs. A library for SVM called LIBSVM toolbox in the MATLAB environment is employed with a radial basis kernel function K(*x*, *xj*) = $e^{-\gamma {\left|x-x_{j}\right|}^{2}},$ where γ = 0.2 [35].

### 3.4. Least Squares Method 

The LSM algorithm needs two average reference vectors calculated for falls and ADLs in order to come up with a class decision [34]. For a given test vector $x={\left[x_{1},\ldots ,x_{m}\right]}^{T}$ sum square errors, $\varepsilon_{i}^{2}$, between the reference vectors $r_{i}={\left[r_{i1},\ldots ,r_{iM}\right]}^{T}$, *i* = 1, 2 are calculated. The class decision is given as follows by minimizing $\varepsilon_{i}^{2}$:

(20) $$\varepsilon_{i}^{2}=\sum_{m=1}^{M}{\left(x_{m}-r_{im}\right)}^{2},i=1,2$$

### 3.5. Dynamic Time Warping 

DTW finds optimal alignment between two given time dependence sequences under certain restrictions. In order to achieve a perfect match, the sequences are warped nonlinearly by stretching or compressing in the time domain. Basically, elements between the test vector and reference vectors are calculated using the Euclidean distance as a cost measure. DTW aims to find the least-cost warping path and allows similar shapes to match, even if they are out of phase in the time axis. Because of its adaptive structure, DTW is widely used in many pattern recognition problems, such as speech recognition, signature and gait recognition, fingerprint pairing, face localization in color images and ECG signal classification [36].

### 3.6. Artificial Neural Networks

ANN is one of most preferred classifier models in pattern recognition and classification problems. ANN can be defined as a set of independent processing units, which receive their inputs through weighted connections [31]. In this work, a multilayer ANN, which consists of one input layer, two hidden layers and one output layer, has been used. The input layer has 30 input neurons, and the output layer has one output neuron. In the hidden layers, the sigmoid activation function is used, but in the output neuron, the purelin linear activation function is used. ANN is created by the Neural Networks Toolbox in the MATLAB environment and trained with a back propagation algorithm, namely the Levenberg–Marquardt (LM) algorithm. The class decision is made by the following rule by normalizing data between 0 and 1:

(21) $$OUT=\{\begin{array}{cc} ADL, & OUT\geq 0.5\\ Fall, & OUT<0.5 \end{array}$$

## 4. Results and Discussion

Comparisons of the six machine learning algorithm’s accuracy performances based on sensor combinations are shown in Table 4. When all six sensor nodes are used for classification, the *k*-NN algorithm gives 99.91% accuracy; however, the best accuracy (99.94%) is achieved using three different sensor combinations, which are ECA, FDBA and FECA, right-thigh_waist_head, right-ankle_right-wrist_chest_head and right-ankle_right-thigh-waist-head, respectively. Increasing the number of sensor nodes does not guarantee the best classification results. When the single-sensor results are examined, it is clear that the waist sensor, labeled C, gives alone the best accuracy (99.87%) with the *k*-NN algorithm. 

The BDM algorithm accuracy performances are promising and satisfactory. BDM produces 99.26% classification accuracy when six sensor units are used in the calculation; however, the best accuracy (99.90%) is achieved with the ECA sensor combination (right-thigh_waist_head). The waist sensor, labeled C, gives 99.24% accuracy itself.

SVM is known as a very robust classifier, and its classification accuracy performances are also very good in this work. SVM classification accuracy is 99.48% when all six sensor nodes are used; however, the best accuracy (99.69%) is achieved with FEA and FEBA sensor combinations, which are right-ankle_right-thigh_head and right-ankle_right-thigh_chest_head, respectively. When single sensor results are examined, it is clear that the right-thigh sensor, labeled E, gives alone the best accuracy (99.27%) with the SVM algorithm.

The LSM algorithm has a very simple structure in terms of computation, and this characteristic makes it advantageous in embedded hardware implementations. The LSM algorithm accuracy performance is also good, but the sensitivity of the algorithm is decreased compared to the *k*-NN, BDM and SVM algorithms. When all six sensor nodes are used, then LSM gives 99.65% accuracy; however, the best accuracy (99.67%) is achieved with the FEBA and FEDA sensor combinations, right-ankle_right-thigh_chest_head and right-ankle_right-thigh_right-wrist_head, respectively. When single sensor behaviors are taken into consideration, it is clear that the waist sensor, labeled C, gives alone the best result, with 98.46% accuracy.

DTW produces 97.85% accuracy with all six sensor nodes are used in the classification process. However, the best accuracy (98.67%) is achieved with a quintuple sensor combination (EDCBA), which is right-thigh_right-wrist_waist_chest_head. The waist sensor, labeled C, gives 98.29% accuracy itself, and this performance is the best result for DTW.

When all six sensor nodes are used, then ANN gives 95.68% accuracy; however, the best accuracy (96.27%) is achieved with the ECBA sensor combination, that is right-thigh_waist_chest_head. When single-sensor results are investigated from the table, the waist sensor, labeled C, is found to be the most accurate, with the accuracy of 95.68%. 

The best results of the double, triple, quadruple and quintuple sensor configurations are given under the individual machine learning algorithm captions in Table 5. The accuracies of all of these sensor configurations are over 99% accuracy for *k*-NN, BDM, SVM and LSM algorithms. It is clearly seen from the table that the fall detection performances are gradually decreased for the DTW and ANN algorithms. Perfect sensitivity performance is achieved at a 100% *Se* rate both with the FDBA (right-ankle _right-wrist_chest_head) quadruple sensor configuration using the *k*-NN algorithm and the FEDCA (right-ankle_right-thigh_right-wrist_waist_head) quintuple sensor configuration using the LSM algorithm. The best performance of this work is achieved with the FDBA configuration using the *k*-NN algorithm (100% *Se*, 99.89% *Sp* and 99.94% *Acc*); this result is even better than the FEDCBA configuration (100% Se, 99.84 *Sp* and 99.91% *Acc*) in which all sensor units are used. 

This work proves that it is possible to achieve very high accuracies with single-sensor options in fall detection applications. There is no strong relation between the number of sensor units and classification performance, because increasing the number of sensor units slightly improves the accuracy. Table 6 summaries single sensor accuracies for six machine learning techniques, and almost all of them are higher than 95%. The best single-sensor performance (99.96% sensitivity, 99.87% accuracy and 99.76% specificity) is achieved with the waist sensor using the *k*-NN algorithm. 99.96% sensitivity means a very good performance, because there are only six false negative; in other words, six missed falls exist in 10 rounds of the whole dataset. The dataset consists of 2520 movements and 1400 of them are falls. When the classifier runs in 10 rounds, then 14,000 falls are evaluated by the classification algorithm. The *k*-NN algorithm detects 13,994 falls out of 14,000, and this implies that the algorithm reaches 100% sensitivity in some rounds (see Table 7). 

When single sensor solutions’ results are investigated, it is clearly seen that the best performance (99.87% accuracy) is achieved with the waist sensor, labeled C, by using the *k*-NN algorithm, and the average of accuracy of six machine learning techniques for this sensor (waist) is 98.42%. The waist sensor is the closest unit to the trunk. Thus, it is not affected much by interpersonal differences in the body movement of subjects. This immunity to position-based interpersonal difference enables better performance than from the outer limbs. The second best performance (97.89% accuracy) is achieved with the right-thigh sensor, labeled E, as an average accuracy of six sensor units. The right-ankle sensor, labeled F, is the third best sensor placement part of the body and produces 97% average accuracy. The head sensor, labeled A, gives 96.61% average accuracy, and this is the fourth best performance. Despite the fact that the chest sensor, labeled B, is close to the trunk, similar to the waist and thigh sensors, its average of accuracy is only 96.50%. The reason for this lack of performance is because of the anatomical interpersonal differences. The position of the chest sensor varies depending on the subjects’ gender, posture and physical characteristics, such as obesity, thinness, etc. This causes an increase in interpersonal differences and indirectly decreases the accuracy performances of fall detection. The worst performance is observed with the right-wrist sensor, labeled D, with 94.97% average accuracy; because this outer limb is the location where interpersonal differences (behavior and act) become evident. Whereas the wrist is the highly preferred body location for wearable devices currently, this location is not suitable for fall detection applications. Average sensor accuracies discussed in this section are given in Table 8.

**Figure 6:**
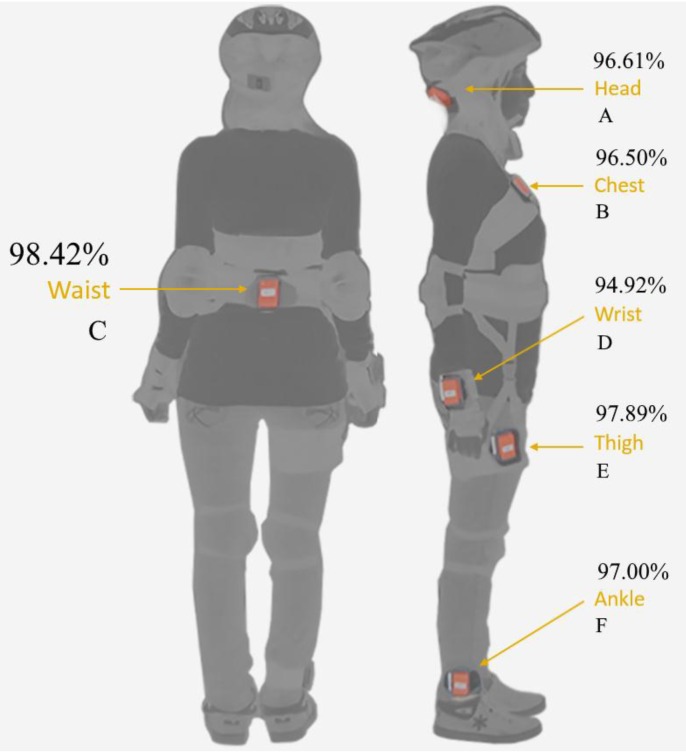
Location-based average accuracies.

Location-based average accuracies for single sensor units are visualized in Figure 6. The waist sensor unit gives the best accuracy performances with all machine learning algorithms used in this study, except SVM (see Table 6). The average accuracy of the waist sensor is 98.42% for six machine learning algorithms, and this is the best average accuracy. The second best average accuracy performance is achieved with the thigh sensor unit at a rate of 97.89% accuracy. The third best average accuracy is 97.00% and achieved with the ankle sensor unit. The fourth is 96.61% and achieved with the head sensor unit. The fifth is 96.50% and achieved with the chest. The wrist sensor unit gives the worst accuracy performances with six machine learning algorithm, except DTW. The average accuracy of the wrist sensor unit is 94.92%. 

The observed performance differences between the body parts give clues about determining the correct sensor placement location on the body. The sensor unit in the waist region is the closest to the center of gravity of the body; this may be the sole reason for the best performance being observed at that location. However, it is understood from the results that using the waist sensor as a reference on calculating the maximum value of TA vector has a very small contribution to the fall detection performance of the waist sensor, as well. It was expected to achieve better accuracies with the head sensor unit, because the head is the region that contains the human vestibular (balance) system. Therefore, the head region anatomically has a critical importance for sensing body movements. It is believed that the reason for having worse performance with the head sensor unit is the dataset used in this work, because the dataset has been created using real-world ADLs, but voluntary (simulated) falls. Hence, even if volunteers make an effort to perform falls more naturally, their body reflexes keep their head from heavy impacts (hits) on the ground due to the autonomic nervous system. 

The right-ankle sensor unit’s fall detection performance was the third best performance after the right-thigh sensor unit performance. In spite of the fact that the ankle is an outer limb, like the wrist, better accuracy was achieved with the right-ankle sensor unit than the right-wrist sensor unit. The reason why using this sensor gives a better classification result can be explained by the fact that the feet have limited motion compared to our hands/arms, as feet carry the body. 

Results can also be analyzed in terms of algorithm performances; the best performance is achieved with the *k*-NN algorithm. The average accuracy of six single sensors for *k*-NN is 99.92%. The BDM algorithm has 97.77% average accuracy, and this is the second best classification performance. The SVM algorithm average accuracy is 97.49%, and this is slightly smaller than BDM; this result has the third best classification performance. The LSM, DTW and ANN algorithms have average accuracies 96.6%, 95.6% and 94.6%, respectively. There is only a 1% performance difference between these three classifiers. The performance summation is given in Table 8, both for sensor location and algorithm, in reverse order. The efficiency of the classification algorithm is more prominent than the sensor location for fall detection performance. As a result, it is possible to create a robust automatic fall detection device using the *k*-NN algorithm with a single sensor unit placed at the waist region of the body. The last section of Table 8 gives the first six best individual sensor performances. The first four best performances are achieved with the *k*-NN algorithm, but different sensor units. In addition, the waist sensor repeats its location-based performance advantage by appearing again in the table as having the sixth best performance with another classifier, BDM. 

This work is uniquely focused on determining the best sensor placement part on the human body for wearable fall detection systems. A dataset consisting of 2520 movements, including 16 types of ADLs and 20 types of falls is created by seven men and seven women with and ethics approval. In this work six sensor units each with a triaxial (accelerometer, gyroscopes and magnetometer) sensor are used on the different parts of the human body. Another important advantage of this work is the variety of machine learning techniques employed in order to distinguish falls from ADLs. To the best of our knowledge, this work is the most comprehensive investigation in the literature about analyzing the fall detection performance of sensor placement parts on the human body for wearable devices.

## 5. Conclusions 

In this paper, a comprehensive analysis of sensor placement locations for the human body over fall detection performance has been made. There are some works in the literature that give ideas about location-based performances [18,19,20]; however, the literature suffers from work that specially focused on sensor location performances [22]. Table 9 summarizes related works in terms of the number of sensors (Sens.), technical specifications of the used sensors (Spec.), volunteers participating in the tests (Vol.), sensor locations on the human body (Locat.), the investigated sensor combinations (Comb.), the number of movement types included in the dataset (Tests), the employed classification algorithms (Algorithms) and the classification performance metrics (Performances). This work has many advantages from the other works; for example in this work, the number of male and female volunteers is equal; however, the datasets used in other works in the literature are male dominated, and this makes their dataset unbalanced. The dataset used in this work contains 36 types of movements, including 16 ADLs and 20 falls. In this work, 378 sensor combinations are investigated. Six machine learning techniques are employed. A 99.96% sensitivity is achieved with a single waist sensor using the *k*-NN algorithm. These advantages show why this work is the most comprehensive investigation in the literature about sensor placement performance on fall detection. 

Accurate fall detection systems in the existing literature generally use multiple sensors. However, the increase in the number of sensor nodes has a negative impact on the adoption of the system by the user. A fall detection system needs to be simple so as not to affect users’ routine daily lives. As a result, the reduction of the number of sensor nodes brings many advantages, which are improved mobility, reduced computational load, decreased power consumption and increased ease of use.

Each sensor unit used in this work is comprised of a triaxial (accelerometer, gyroscope, and magnetometer) sensor. This means nine sensors’ data stream at a time towards the feature extraction unit. This unit creates a feature vector from a 4 s-long frame, which is constructed at a 25-Hz sampling rate around the maximum TA value. The feature vector of an individual sensor unit has 234 elements; therefore, the feature vector belonging to the six sensor units has 1404 elements (234 × 6). While the feature vectors decrease in size from 1404 to 234 by reducing the number of sensor units, the capability of estimating the variance of the feature vectors with the PCA algorithm increases from 72.38% to 96.63% with the same amount of PCs (30 PCs). This situation has a great contribution toward increased accuracy in single-sensor combinations. Therefore, decreasing the number of sensor units may not be considered as a disadvantage. 

The world’s current aged population is not eager to use technology in their daily lives, because they do not feel comfortable with body-attached wearable devices. However, wearable technologies are still one of the most popular fields in today’s entrepreneurship trends. The current young and middle-aged generation is going to be aging and in the near future, we will have an elderly population that will be much more aware of technology than today. This makes wearable technology a more attractive field for the future.

Although this work did not use elderly motion records, it is aimed to test the proposed fall detection system with elderly data in the future. For this purpose, another ethical approval that allows the collection of elderly ADL data was already obtained from the Erciyes University Ethics Committee (Approval Number 2015/411). 

Lastly, to enable a comparison among the algorithms developed in different studies, it is intended to make this dataset publicly available at the University of Irvine Machine Learning Repository [37].

## Figures and Tables

**Table 1:** Age, sex and anthropometric information of volunteers.

	Men							Women							All
**Volunteers**	**101**	**102**	**103**	**104**	**106**	**107**	**108**	**203**	**204**	**205**	**206**	**207**	**208**	**209**	**Ave.**
**Age**	21	21	23	27	22	21	21	21	21	20	19	20	24	22	21.64
**Height (kg)**	75	81	78	67	54	72	68	51	47	51	47	60	55	70	62.57
**Weight (cm)**	170	174	180	176	160	175	184	170	157	169	166	165	163	182	170.79

**Table 2:** Falls and activities of daily living (ADLs) movement list.

Activities of Daily Living (ADLs)			Voluntary Falls		
#	Label	Description	#	Label	Description
**801**	Walking fw	Walking forward	**901**	Front lying	From vertical going forward to the floor
**802**	Walking bw	Walking backward	**902**	Front protected lying	From vertical going forward to the floor with arm protection
**803**	Jogging	Running	**903**	Front knees	From vertical going down on the knees
**804**	Squatting down	Going down, then up	**904**	Front knees lying	From vertical going down on the knees and then lying on the floor
**805**	Bending	Bending of about 90 degrees	**905**	Front quick recovery	From vertical going on the floor and quick recovery
**806**	Bending and pick up	Bending to pick up an object on the floor	**906**	Front slow recovery	From vertical going on the floor and slow recovery
**807**	Limp	Walking with a limp	**907**	Front right	From vertical going down on the floor, ending in right lateral position
**808**	Stumble	Stumbling with recovery	**908**	Front left	From vertical going down on the floor, ending in left lateral position
**809**	Trip over	Bending while walking and then continue walking	**909**	Back sitting	From vertical going on the floor, ending sitting
**810**	Coughing	Coughing or sneezing	**910**	Back lying	From vertical going on the floor, ending lying
**811**	Sit chair	From vertical sitting with a certain acceleration on a chair (hard surface)	**911**	Back right	From vertical going on the floor, ending lying in right lateral position
**812**	Sit sofa	From vertical sitting with a certain acceleration on a sofa (soft surface)	**912**	Back left	From vertical going on the floor, ending lying in left lateral position
**813**	Sit air	From vertical sitting in the air exploiting the muscles of legs	**913**	Right sideway	From vertical going on the floor, ending lying
**814**	Sit bed	From vertical sitting with a certain acceleration on a bed (soft surface)	**914**	Right recovery	From vertical going on the floor with subsequent recovery
**815**	Lying bed	From vertical lying on the bed	**915**	Left sideway	From vertical going on the floor, ending lying
**816**	Rising bed	From lying to sitting	**916**	Left recovery	From vertical going on the floor with subsequent recovery
			**917**	Rolling out bed	From lying, rolling out of bed and going on the floor
			**918**	Podium	From vertical standing on a podium going on the floor
			**919**	Syncope	From standing going on the floor following a vertical trajectory
			**920**	Syncope wall	From standing going down slowly slipping on a wall

**Table 3:** Combinations of sensor units on the body. COMB is combinations. A-head, B-chest, C-waist, D-right wrist, E-right thigh and F-right ankle.

	SENSORS								SENSORS						
*	Ankle	Thigh	Wrist	Waist	Chest	Head	COMB	*	Ankle	Thigh	Wrist	Waist	Chest	Head	COMB
	F	E	D	C	B	A			F	E	D	C	B	A	
**0**	0	0	0	0	0	0		**32**	1	0	0	0	0	0	**F**
**1**	0	0	0	0	0	1	**A**	**33**	1	0	0	0	0	1	**AF**
**2**	0	0	0	0	1	0	**B**	**34**	1	0	0	0	1	0	**BF**
**3**	0	0	0	0	1	1	**AB**	**35**	1	0	0	0	1	1	**ABF**
**4**	0	0	0	1	0	0	**C**	**36**	1	0	0	1	0	0	**CF**
**5**	0	0	0	1	0	1	**AC**	**37**	1	0	0	1	0	1	**ACF**
**6**	0	0	0	1	1	0	**BC**	**38**	1	0	0	1	1	0	**BCF**
**7**	0	0	0	1	1	1	**ABC**	**39**	1	0	0	1	1	1	**ABCF**
**8**	0	0	1	0	0	0	**D**	**40**	1	0	1	0	0	0	**DF**
**9**	0	0	1	0	0	1	**AD**	**41**	1	0	1	0	0	1	**ADF**
**10**	0	0	1	0	1	0	**BD**	**42**	1	0	1	0	1	0	**BDF**
**11**	0	0	1	0	1	1	**ABD**	**43**	1	0	1	0	1	1	**ABDF**
**12**	0	0	1	1	0	0	**CD**	**44**	1	0	1	1	0	0	**CDF**
**13**	0	0	1	1	0	1	**ACD**	**45**	1	0	1	1	0	1	**ACDF**
**14**	0	0	1	1	1	0	**BCD**	**46**	1	0	1	1	1	0	**BCDF**
**15**	0	0	1	1	1	1	**ABCD**	**47**	1	0	1	1	1	1	**ABCDF**
**16**	0	1	0	0	0	0	**E**	**48**	1	1	0	0	0	0	**EF**
**17**	0	1	0	0	0	1	**AE**	**49**	1	1	0	0	0	1	**AEF**
**18**	0	1	0	0	1	0	**BE**	**50**	1	1	0	0	1	0	**BEF**
**19**	0	1	0	0	1	1	**ABE**	**51**	1	1	0	0	1	1	**ABEF**
**20**	0	1	0	1	0	0	**CE**	**52**	1	1	0	1	0	0	**CEF**
**21**	0	1	0	1	0	1	**ACE**	**53**	1	1	0	1	0	1	**ACEF**
**22**	0	1	0	1	1	0	**BCE**	**54**	1	1	0	1	1	0	**BCEF**
**23**	0	1	0	1	1	1	**ABCE**	**55**	1	1	0	1	1	1	**ABCEF**
**24**	0	1	1	0	0	0	**DE**	**56**	1	1	1	0	0	0	**DEF**
**25**	0	1	1	0	0	1	**ADE**	**57**	1	1	1	0	0	1	**ADEF**
**26**	0	1	1	0	1	0	**BDE**	**58**	1	1	1	0	1	0	**BDEF**
**27**	0	1	1	0	1	1	**ABDE**	**59**	1	1	1	0	1	1	**ABDEF**
**28**	0	1	1	1	0	0	**CDE**	**60**	1	1	1	1	0	0	**CDEF**
**29**	0	1	1	1	0	1	**ACDE**	**61**	1	1	1	1	0	1	**ACDEF**
**30**	0	1	1	1	1	0	**BCDE**	**62**	1	1	1	1	1	0	**BCDEF**
**31**	0	1	1	1	1	1	**ABCDE**	**63**	1	1	1	1	1	1	**ACBDEF**

**Table 4:** Machine learning algorithms’ performances based on sensor combinations. COMB is combinations, A-head, B-chest, C-waist, D-right wrist, E-right thigh and F-right ankle. *k*-NN, *k*-nearest neighbor classifier; BDM, Bayesian decision making; SVM, support vector machines; LSM, least squares method; DTW, dynamic time warping; ANN, artificial neural network.

No.	COMB	*k*-NN	BDM	SVM	LSM	DTW	ANN	No.	COMB	*k*-NN	BDM	SVM	LSM	DTW	ANN
0	NONE							32	F	99.50	98.24	99.06	96.36	93.51	95.30
1	A	99.20	97.29	96.08	96.77	96.12	94.20	33	FA	99.91	99.19	99.35	99.04	97.04	95.56
2	B	99.60	96.65	96.28	95.53	96.58	94.35	34	FB	99.55	98.85	99.22	98.27	96.31	95.13
3	BA	99.69	98.74	98.19	98.10	97.12	95.29	35	FBA	99.70	99.43	99.32	99.32	97.04	95.61
**4**	**C**	**99.87**	**99.24**	**98.99**	**98.46**	**98.29**	**95.69**	36	FC	99.81	99.47	99.59	98.66	96.24	95.46
5	CA	99.92	99.60	99.37	98.88	97.11	95.67	37	FCA	99.93	99.74	99.55	99.38	97.41	95.55
6	CB	99.77	99.23	99.00	98.13	97.35	95.63	38	FCB	99.80	99.34	99.42	99.02	97.30	95.58
7	CBA	99.76	99.54	99.37	99.17	96.84	95.82	39	FCBA	99.83	99.53	99.62	99.59	97.52	95.70
8	D	97.49	96.08	95.27	94.63	93.62	92.40	40	FD	99.60	97.88	98.92	98.35	95.63	94.50
9	DA	99.65	98.52	96.72	98.56	96.99	94.29	41	FDA	99.82	98.65	98.97	99.48	97.35	95.13
10	DB	99.54	98.24	97.63	96.38	94.42	94.23	42	FDB	99.79	98.69	98.71	98.60	95.25	95.14
11	DBA	99.76	98.57	98.04	98.73	96.75	95.42	43	FDBA	99.94	99.29	99.07	99.35	97.17	95.77
12	DC	99.54	98.67	98.84	98.70	97.19	94.95	44	FDC	99.77	98.91	99.38	99.12	96.60	95.16
13	DCA	99.83	99.29	99.02	99.29	97.09	95.53	45	FDCA	99.92	99.08	99.47	99.54	98.19	95.38
14	DCB	99.66	98.75	98.82	98.60	95.86	95.08	46	FDCB	99.85	98.85	99.16	99.00	97.41	95.31
15	DCBA	99.80	99.06	99.23	99.13	97.35	95.39	47	FDCBA	99.85	99.16	99.37	99.48	97.40	95.92
16	E	99.61	99.12	99.27	98.09	95.69	95.53	48	FE	99.75	99.15	99.57	98.18	94.79	95.54
17	EA	99.81	99.13	99.52	98.39	97.37	95.53	49	FEA	99.91	99.63	99.69	99.15	96.76	95.47
18	EB	99.82	99.19	99.55	98.74	96.58	95.71	50	FEB	99.70	99.49	99.47	99.00	94.95	95.46
19	EBA	99.79	99.64	99.58	99.21	97.88	96.02	51	FEBA	99.78	99.79	99.69	99.67	97.92	95.77
20	EC	99.84	99.44	99.31	98.82	95.85	95.17	52	FEC	99.88	99.67	99.64	99.07	96.65	95.58
21	ECA	99.94	99.90	99.59	98.93	97.88	95.83	53	FECA	99.94	99.73	99.66	99.52	97.66	95.59
22	ECB	99.91	99.42	99.37	99.32	96.85	95.60	54	FECB	99.86	99.51	99.53	99.44	97.53	95.59
23	ECBA	99.88	99.67	99.62	99.31	98.11	96.27	55	FECBA	99.86	99.65	99.67	99.56	97.40	96.18
24	ED	99.63	98.60	99.29	98.41	95.62	94.93	56	FED	99.70	98.97	99.55	99.08	96.69	95.18
25	EDA	99.82	99.00	99.42	98.45	97.86	95.68	57	FEDA	99.87	99.17	99.45	99.67	98.11	95.42
26	EDB	99.77	99.04	99.38	98.77	96.41	95.30	58	FEDB	99.83	99.25	99.21	99.25	97.27	95.36
27	EDBA	99.87	99.25	99.31	99.27	97.53	95.58	59	FEDBA	99.90	99.30	99.38	99.48	98.02	95.63
28	EDC	99.69	99.09	99.35	99.00	97.49	95.30	60	FEDC	99.82	99.09	99.50	99.22	96.46	95.42
29	EDCA	99.88	99.22	99.56	99.45	96.69	95.72	61	FEDCA	99.88	99.28	99.59	99.57	98.19	95.62
30	EDCB	99.84	99.05	99.40	99.13	96.72	95.51	62	FEDCB	99.87	99.13	99.34	99.37	97.09	95.67
31	EDCBA	99.86	99.21	99.39	99.33	98.67	95.75	63	FEDCBA	99.91	99.26	99.48	99.65	97.85	95.68

**Table 5:** The best results of respective sensor combinations for double, triple, quadruple and quintuple. (P: positive; N: negative). A-head, B-chest, C-waist, D-right wrist, E-right thigh and F-right ankle. Acc (%)* is accuracy.

			CONFUSION MATRICES																
			*k*-NN			BDM			SVM			LSM			DTW			ANN	
**Double**			P	N		P	N		P	N		P	N		P	N		P	N
	TRUE	P	1398	2		1398.5	1.5		1395.2	4.8		1387.3	12.7		1372.5	27.5		1356.6	43.4
		N	0	1120		8.6	1111.4		5.6	1114.4		11.4	1108.6		38.7	1081.3		64.6	1055.4
Acc (%)*			**99.92**			**99.60**			**99.59**			**99.04**			**97.37**			**95.71**	
**Combinations**			**CA**			**CA**			**FC**			**FA**			**EA**			**EB**	
**Triple**			P	N		P	N		P	N		P	N		P	N		P	N
	TRUE	P	1399	1		1399	1		1395.1	4.9		1397	3		1384	16		1366.1	33.9
		N	0.4	1119.6		1.4	1118.6		3	1117		10.2	1109.8		37.4	1082.6		66.5	1053.5
Acc (%)*			**99.94**			**99.90**			**99.69**			**99.48**			**97.88**			**96.02**	
**Combinations**			**ECA**			**ECA**			**FEA**			**FDA**			**EBA**			**EBA**	
**Quadruple**			P	N		P	N		P	N		P	N		P	N		P	N
	TRUE	P	1400	0		1398.3	1.7		1395.4	4.6		1399.1	0.9		1381	19		1368.5	31.5
		N	0.5	1118.5		3.6	1116.4		3.1	1116.9		7.3	1112.7		26.7	1093.3		62.4	1057.6
Acc (%)*			**99.94**			**99.79**			**99.69**			**99.67**			**98.19**			**96.27**	
**Combinations**			**FDBA**			**FEBA**			**FEBA**			**FEDA**			**FDCA**			**ECBA**	
**Quintuple**			P	N		P	N		P	N		P	N		P	N		P	N
	TRUE	P	1399.7	0.3		1398.2	1.8		1394.8	5.2		1400	0		1389.4	10.6		1367.6	32.4
		N	2.2	1117.8		7	1113		3	1117		10.9	1109.1		22.9	1097.1		63.8	1056.2
Acc (%)*			**99.90**			**99.65**			**99.67**			**99.57**			**98.67**			**96.18**	
**Combinations**			**FEDBA**			**FECBA**			**FECBA**			**FEDCA**			**EDCBA**			**FECBA**	

**Table 6:** Comparison of the single sensor unit’s fall detection performances with different machine learning techniques. A-head, B-chest, C-waist, D-right wrist, E-right thigh and F-right ankle. Acc (%)* is accuracy.

		CONFUSION MATRICES											
		*k*-NN		BDM		SVM		LSM		DTW		ANN	
**C (Waist)**		P	N	P	N	P	N	P	N	P	N	P	N
TRUE	P	1399.4	0.6	1396.2	3.8	1391.7	8.3	1395.2	4.8	1385.6	14.4	1359.1	40.9
	N	2.7	1117.3	15.3	1104.7	17.1	1102.9	34	1086	28.6	1091.4	67.8	1052.2
Acc (%)*		**99.87**		**99.24**		**98.99**		**98.46**		**98.29**		**95.69**	
**E (Thigh)**		P	N	P	N	P	N	P	N	P	N	P	N
TRUE	P	1395.2	4.8	1390.7	9.3	1395	5	1371.5	28.5	1320.4	79.6	1354.2	45.8
	N	5	1115	12.8	1107.2	13.4	1106.6	19.7	1100.3	28.9	1091.1	66.8	1053.2
Acc (%)*		**99.61**		**99.12**		**99.27**		**98.09**		**95.69**		**95.53**	
**F (Ankle)**		P	N	P	N	P	N	P	N	P	N	P	N
TRUE	P	1392.6	7.4	1390.6	9.4	1389.2	10.8	1326.6	73.4	1273.1	126.9	1358.8	41.2
	N	5.2	1114.8	34.9	1085.1	12.8	1107.2	18.3	1101.7	36.6	1083.4	77.3	1042.7
Acc (%)*		**99.5**		**98.24**		**99.06**		**96.36**		**93.51**		**95.3**	
**A (Head)**		P	N	P	N	P	N	P	N	P	N	P	N
TRUE	P	1391	9	1384.6	15.4	1372.3	27.7	1376.5	23.5	1362.2	37.8	1354.4	45.6
	N	11.1	1108.9	52.9	1067.1	71	1049	57.9	1062.1	60	1060	100.6	1019.4
**ACC**		**99.2**		**97.29**		**96.08**		**96.77**		**96.12**		**94.2**	
**B (Chest)**		P	N	P	N	P	N	P	N	P	N	P	N
TRUE	P	1398.1	1.9	1380.8	19.2	1363.9	36.1	1388.6	11.4	1381.4	18.6	1341.1	58.9
	N	8.1	1111.9	65.3	1054.7	57.6	1062.4	101.3	1018.7	67.5	1052.5	83.5	1036.5
Acc (%)*		**99.6**		**96.65**		**96.28**		**95.53**		**96.58**		**94.35**	
**D (Wrist)**		P	N	P	N	P	N	P	N	P	N	P	N
TRUE	P	1370.7	29.3	1371.9	28.1	1353.8	46.2	1302.7	97.3	1314.2	85.8	1343	57
	N	33.9	1086.1	70.8	1049.2	73.1	1046.9	37.9	1082.1	75.1	1044.9	161.6	985.4
Acc (%)*		**97.49**		**96.08**		**95.27**		**94.63**		**93.62**		**92.4**	

**Table 7:** *k*-NN classifier results over 10 successive rounds with the waist (C) sensor unit. AVG: average; STD: standard deviation.

Run	1	2	3	4	5	6	7	8	9	10	AVG	STD
*Se* (%)	99.93	99.93	100	99.93	99.93	100	99.93	100	100	99.93	99.96	0.0369
*Acc* (%)	99.88	99.88	99.92	99.76	99.88	99.92	99.88	99.84	99.84	99.88	99.87	0.0460
*Sp* (%)	99.82	99.82	99.82	99.55	99.82	99.82	99.82	99.64	99.64	99.82	99.76	0.1035
*TN*	1118	1118	1118	1115	1118	1118	1118	1116	1116	1118	1117.3	1.1595
*FP*	2	2	2	5	2	2	2	4	4	2	2.7	1.1595
*TP*	1399	1399	1400	1399	1399	1400	1399	1400	1400	1399	1399.4	0.5164
*FN*	1	1	0	1	1	0	1	0	0	1	0.6	0.5164

**Table 8:** Summary of the location and algorithm-based accuracy averages and individual sensor performances, calculated from Table 6. A-head, B-chest, C-waist, D-right wrist, E-right thigh and F-right ankle.

**Location of Sensor Unit**	**C (Waist)**	**E (Right-Thigh)**	**F (Right-Ankle)**	**A (Head)**	**B (Chest)**	**D (Right-Wrist)**
**Average Accuracy (%)**	98.42	97.89	97.00	96.61	96.50	94.92
						
**Algorithm**	***k*-NN**	**BDM**	**SVM**	**LSM**	**DTW**	**ANN**
Average Accuracy (%)	99.21	97.77	97.49	96.64	95.64	94.58
						
**Individual Sensor Unit**	**C (Waist)**	**E (Right-Thigh)**	**B (Chest)**	**A (Head)**	**E (Right-Thigh)**	**C (Waist)**
	***k*** **-NN**	***k*** **-NN**	***k*** **-NN**	***k*** **-NN**	**SVM**	**BDM**
Accuracy (%)	99.87	99.61	99.60	99.50	99.27	99.24

**Table 9:** Comparison of the literature works. Sens., sensor; Spec., specification; Vol., volunteer; Locat., location.

	Sens.	Spec.	Vol.	Locat.	Comb.	Tests	Algorithms	Performances
Bao [21]	5× 2X A	±10 g	20 P 13 M 7 F	ankle arm thigh hip wrist	20	20 20 ADL 0 fall	Decision Table Instant Learning Naïve Bayes Decision Tree	All Sensors 84.5% *Acc* thigh + wrist 80.73% *Acc*
Kangas [18]	3× 3X A	±12 g	3 P 2 M 1 F	waist head wrist	24	12 9 ADL 3 fall	Rule Based Alg.	98% Head *Se* 97% Waist *Se* 71% Wrist *Se*
Li [19]	2× 3X A 3X G	±10 g ±500°/s	3 P 3 M 0 F	chest thigh	1	14 9 ADL 5 fall	Rule Based Alg.	chest + thigh 92% *Acc* 91% *Se*
Atallah [20]	6× 3X G	±3 g	11 P 9 M 2 F	ankle knee waist wrist arm chest ear	12	15 15 ADL	*k*-NN Bayesian Classifier	low level waist medium level chest, wrist high level arm, knee
Shi [22]	21× 3X A 3X G 3X M	±8 g ±2000°/s N/A	13 P 12 M 1 F	thighs shanks feet u-arms f-arms hands waist neck head back	14	25 12 ADL 13 fall	Decision Tree Algorithm	waist 97.79% *Acc* 95.5% *Se* 98.8% *Sp*
Özdemir [13]	6× 3X A 3X G 3X M	±16 g ±1200°/s ±1.5 G	14 P 7 M 7 F	head chest waist wrist thigh knee	378	36 16 ADL 20 fall	*k*-NN BDM SVM LSM DTW ANN	waist 99.87% *Acc* 99.96% *Se* 99.76% *Sp*

In the sensors column, 6× means the number of sensor nodes is 6, 3X A means 3-axis accelerometer, 3X G means 3-axis gyroscope and 3X M means 3-axis magnetometer. In the volunteer column, P, M, and F mean people, male and female respectively.
